# Bat Community Response to Insect Abundance in Relation to Rice Phenology in Peninsular Malaysia

**DOI:** 10.3390/biology15010069

**Published:** 2025-12-30

**Authors:** Nur-Izzati Abdullah, Nurul-‘Ain Elias, Nobuhito Ohte, Christian E. Vincenot

**Affiliations:** 1 Department of Social Informatics, Graduate School of Informatics, Kyoto University, Yoshidahonmachi, Sakyo-ku, Kyoto 606-8501, Japan; nurizzatiabdullah15@gmail.com (N.-I.A.); nobu@i.kyoto-u.ac.jp (N.O.); 2 Island Bat Research Group, Kyoto 606-8501, Japan; 3 School of Biological Sciences, Universiti Sains Malaysia (USM), Gelugor 11800, Penang, Malaysia; 4 Complex Systems Group (NEXUS::CSR), Faculty of Science, Technology and Medicine, University of Luxembourg, L-4364 Esch-sur-Alzette, Luxembourg

**Keywords:** biodiversity conservation, biological control, insect pest controller, limestone karst, rice fields

## Abstract

Bats provide essential ecosystem services, particularly in regulating insect populations within agricultural landscapes. However, research on bat–insect interactions in Malaysian rice fields remains limited. This study examined bat diversity and insect abundance in the rice field area of Gunung Keriang, Kedah, using two harp traps, two mist nets and a light trap at three selected sites. A total of 2499 bats representing 27 species were captured, alongside 161,539 insects from 11 orders. *Rhinolophus pusillus* was the dominant bat species across all seasons. During the dry season, *Chilo polychrysus* (stem borer) was the dominant insect pest, whereas *Nilaparvata lugens* (brown planthopper) prevailed during the wet season. Insects from the order Coleoptera, dominant in the dry season, are likely consumed by larger bat species with stronger bite forces. Bat foraging activity peaked concurrently with insect emergence, corresponding to periods of high food availability. Statistical analysis revealed significant associations between bat activity, temperature, and rainfall, but not insect abundance. These findings underscore the ecological importance of bats as natural pest regulators in rice field ecosystems and highlight their potential role in promoting sustainable, ecosystem-based agricultural management.

## Simple Summary

Bats are among the most important natural pest controllers in agricultural landscapes. By feeding on insects, they help reduce pest populations that can damage crops and lower yields. Despite their importance, studies on how bats interact with insect populations in Malaysian rice fields are still scarce. In this study, we explored the diversity and activity of bats in relation to insect abundance at Gunung Keriang, Kedah, across different rice-growing seasons. Using harp traps, mist nets and light traps, we recorded 27 bat species and 11 insect orders. The most common bat was *Rhinolophus pusillus*, while *Chilo polychrysus* (stem borer) and *Nilaparvata lugens* (brown planthopper) were the dominant insect pests during the dry and wet seasons, respectively. Bat activity was highest during the same periods when insect activity peaked, suggesting that bats respond to nightly food availability. However, their activity was more strongly influenced by temperature and rainfall than by insect abundance alone. These findings highlight the essential ecological role of bats in naturally regulating pest populations. Encouraging bat-friendly farming practices such as reducing pesticide use and conserving roosting habitats can support sustainable rice production and strengthen ecosystem balance in agricultural areas.

## 1. Introduction

Throughout the paleotropics, bats are often seen as a nuisance by the public [1] and even more so by farmers [2], sometimes even leading to nationally coordinated actions to remove them [3,4]. Yet, bats provide us with important ecosystem services as pollinators and seed dispersers [5,6] and also by regulating insect populations [7]. Thus, in many cases, they could represent a natural ally for agriculture.

Locusts, planthoppers, leafhoppers, and rice stem borers are common insect pests in rice fields [8,9]. Chemical, physical and biological controls are the main options for pest control in agricultural areas, especially in rice fields. The usage of chemicals such as herbicides, rodenticides and insecticides has been proven to have a long-term effect on the ecosystem [10].

Biological controls could also be a good option since they are more efficient as all pests have their own natural enemies [11]. However, this approach has long been neglected, probably due to either ignorance or to farmers preferring immediate results. Barn owls (*Tyto alba*) have been infrequently used to control rat populations in rice fields [12,13]. Using insects that are natural predators of insect pests is another example of biological control. Ladybugs, ants, spiders, dragonflies and mirids [11,14], as well as some vertebrates like frogs, birds, and bats [15], can control the insect pest population naturally. Since rice fields have become the foraging areas for bats, they can potentially be a biological control for insect pests [16].

Insect-eating bats play a role as a natural predator in safeguarding the surrounding protected areas as a biological control [16]. About 70% of bats are insectivorous [17]. Caves serve as an essential roosting site for many bat species all around the world. In caves, bats often roost in large colonies, which can result in the large-scale depletion of insects, especially insect pests in the surrounding agricultural area.

The contribution of bats towards controlling agricultural pests has been mostly documented in the United States [18,19], some parts of Canada [20], Europe [21] and Madagascar [22]. In Southeast Asia, Thailand [15,23,24] has the highest reported studies of bats in rice field areas. However, the situation remains unclear in Malaysia and other Southeast Asian countries. Studies on bats in Malaysian rice field areas are still lacking and more information is needed to determine their contribution to the ecosystem.

The objective of this study is to document insect abundance and insect pest availability across different paddy growth phases during both the dry and wet seasons in rice fields surrounding Gunung Keriang. To date, there remains a lack of data on insect abundance, particularly pest species, across the various stages of rice cultivation in this region. In addition to providing detailed documentation of the insect community, this study also highlights the potential ecological role of insectivorous bats in contributing to natural pest suppression within rice fields. While we did not directly analyze bat diets in this study, previous research has shown that many bat species in Southeast Asian agroecosystems commonly consume agricultural pest taxa, suggesting a plausible link between bat foraging activity and pest availability in the landscape.

## 2. Materials and Methods

### 2.1. Study Area

Gunung Keriang is a small limestone hill rising from a vast rice field landscape north of Alor Setar in northern Peninsular Malaysia. Sampling was conducted in two different paddy growing seasons based on the irrigation schedule of the Muda Agricultural Development Authority (MADA), a regional agency responsible for managing irrigation and agricultural development in the Muda rice granary area.

Season 1—dry season (started in April 2021 and ended in September 2021)
Season 2—wet season (started in October 2021 and ended in February 2022)

Three identified sites were chosen around Gunung Keriang: MADA A (6°10′59.2″ N, 100°19′22.4″ E), MADA B (6°11′57.4″ N, 100°19′50.0″ E), and MADA C (6°11′22.4″ N, 100°20′30.2″ E) based on accessibility, representation of surrounding rice field habitats, and proximity to the limestone hill, which serves as a known bat roosting area.

The trapping was conducted every month for three consecutive days in MADA A, MADA B and MADA C, starting from April 2021 until February 2022. There were five main phases of paddy growing, which are germination, vegetative, reproductive, ripening and harvesting. For the dry season, the reproductive phase was excluded due to movement restrictions that were implemented during that period. For the wet season, all paddy growth phases were recorded.

### 2.2. Insect Trapping

Insects were sampled using light traps that were placed at the three identified sites before sunset. Each light trap used 60 W 12 V tungsten bulbs and was set more than 1 km apart from the others within a 2 km radius from Gunung Keriang, where the harp traps were located. Insect collection was conducted every hour starting at 19:00 until 07:00. Although full insect sampling continued until morning (07:00), only the 19:00–22:00 time window was used in subsequent figures to allow direct comparison with bat capture activity, which is restricted to the same early-night period due to harp trap operation times. This temporal alignment ensures consistency between datasets and prevents misinterpretation arising from differing sampling durations.

The trapped insects were killed by using ethyl acetate; following this, they were sorted and identified to order or family level based on Salleh (1990) [25] and Borror et al. (1996) [26]. Selected insect pests, chosen based on their known status as economically important rice field pests following previous studies and local agricultural records, were identified to species and then stored in 97% alcohol for future molecular analysis. These selections aimed to focus on species of ecological and agricultural relevance rather than representing the full insect diversity in the fields.

### 2.3. Bat Trapping

Insectivorous bats were captured using two harp traps deployed at three relative elevations of Gunung Keriang (top ≈ 200 m a.s.l., middle ≈ 100 m a.s.l. and low ≈ 20 m a.s.l.). Additionally, two mist nets were also set up in the surrounding rice field area. The trapping was conducted for three consecutive nights per sampling session. Mist nets were set up at dusk (19:00) at heights ranging from 5 to 7 m above the ground, were checked every 10 to 15 min and were closed at 22:00. Harp traps were set up at midnight (00:00), were checked every hour and were closed just after dawn (07:00). Captured bats were weighed, sexed and identified based on their morphology [27]. Although harp traps and mist nets are effective for capturing many forest- and edge-adapted insectivorous bats, we acknowledge that these methods may underrepresent highly maneuverable, high-flying or trap-avoidant species. Therefore, capture-based activity reflects primarily the activity of species that are susceptible to these trapping techniques rather than total bat activity across the entire community.

### 2.4. Local Weather Monitoring

The total monthly rainfall, plus the maximum and minimum monthly temperature were provided by the Malaysian Meteorology Department for Alor Setar, Kedah.

### 2.5. Data Analysis

The peak time of insect emergence was analyzed based on the number of insects collected per hour, to examine potential temporal overlap with bat foraging activity. Insect presence was then used to estimate food availability for bats based on different stages of paddy growth (germination, vegetative, reproductive, ripening and harvesting). Insect data were recorded and summarized in two parameters; relative abundance (number of insects of specific taxa/total number of insects) and absolute abundance (number of insects of specific taxa). All the statistical analyses were calculated in R version 4.2.2. The Pearson parametric correlation test was used for the correlation analysis. The variables were first centered and scaled before running the statistical test.

Multiple linear regression was used to examine the relationship between bat activity (measured as the total number of captures per sampling night; dependent variable) and minimum temperature and rainfall (independent variables). Bat activity data were pooled across the three sites for each sampling night and monthly activity totals were used so that the temporal structure of the dataset (i.e., variation among sampling dates) was represented by the corresponding monthly weather variables. Because trapping effort was standardized across sites, location was not included as a predictor in the models.

The regression analysis was also conducted separately for the dominant bat species: *Rhinolophus pusillus*, *Myotis ridleyi*, *Hipposideros larvatus*, *Rhinolophus malayanus* and *Rhinolophus refulgens* using their species-specific capture totals per sampling night as the activity variable. Given the limited number of sampling nights relative to the number of predictors, species-specific regression analyses were treated as exploratory and results were interpreted with caution to minimize the risk of overfitting. All assumptions required for multiple linear regression were assessed using the *check_model()* function from the {performance} R package [28] to ensure the model validity and interpretability.

## 3. Results

### 3.1. Insects

**Figure 1:**
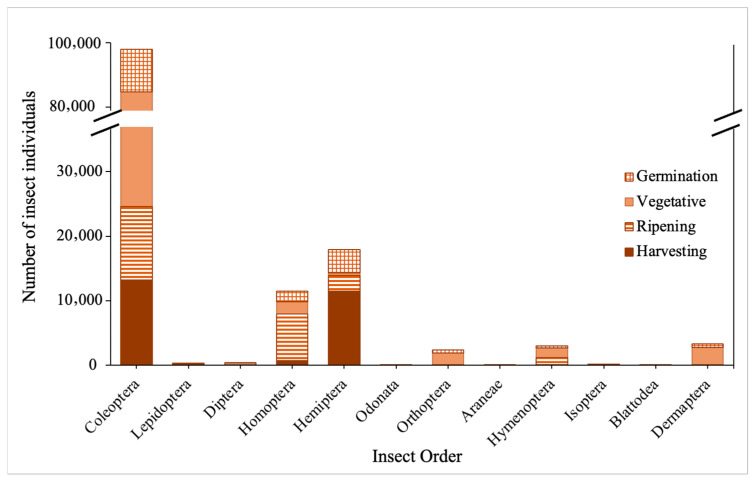
Number of insect individuals captured by order across different growing phases of paddy in the dry season.

For the dry season, 12 orders of insects were recorded at the rice fields (Figure 1) and the dominant insect order was Coleoptera. The germination phase of the paddy had the highest insect abundance compared to the rest of the paddy phases. *Chilo polychrysus* (stem borer) from the order Lepidoptera is the insect pest species that was captured for all phases of paddy growth in the dry season.

**Figure 2:**
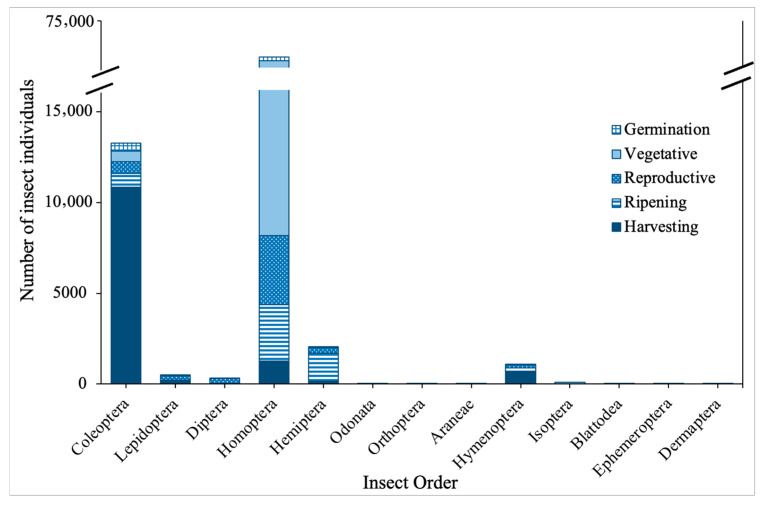
Number of insect individuals captured by order across different growing phases of paddy in the wet season.

In the wet season, 13 orders of insects were also recorded at the rice fields (Figure 2). The dominant insect order for this season was Homoptera. The vegetative phase recorded the highest insect abundance compared to the rest of the paddy phases. *Nilaparvata lugens* (brown planthopper) from the order Hemiptera is the insect pest species that was captured for all phases of paddy growth in the wet season.

Insects from the orders Araneae and Odonata have also been captured in the dry and wet season. These two insect orders are known to be natural enemies of insect pests in rice fields. From the order Araneae, *Lycosa pseudoannulata* was the most common species captured and for the order Odonata, *Agriocnemis* sp. was the most common genus captured.

**Figure 3:**
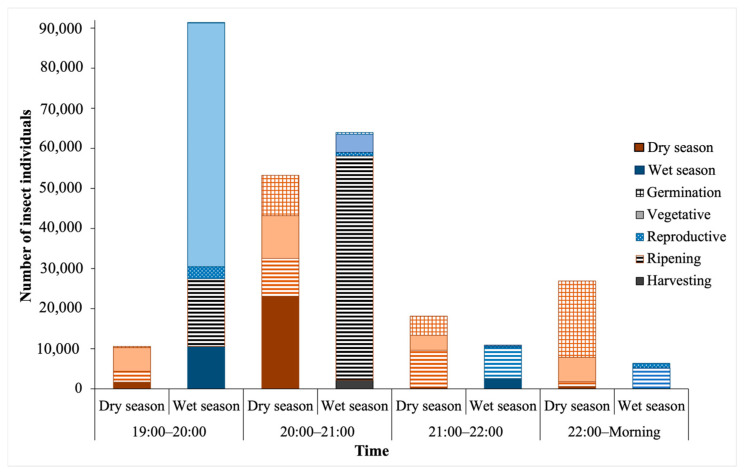
Overall time of insect emergence across different growing phases of paddy in the dry and wet season.

The peak time of insect emergence with the highest number of individuals captured during the dry season, was from 20:00 until 21:00. In contrast, during the wet season, it occurred from 19:00 until 20:00 (Figure 3). This time frame potentially overlap with the peak bat foraging activity.

### 3.2. Bats

A total of 1608 individual bats from six families and 21 species were captured for the dry season. A total of 891 individuals from five families and 23 species were recorded for the wet season (Table 1). *Rhinolophus pusillus* was recorded as the dominant bat species with the highest capture rate in the dry season as well wet season. Throughout this study, rare bat species defined as those represented by one or two individuals captured across all sampling nights within a season were recorded, including species not previously documented at Gunung Keriang. These bats were detected at specific elevations and habitat interfaces around the study area. During the wet season, *Hipposideros halophyllus* was captured at mid elevation, while *Rhinolophus marshalli* was recorded only at the top elevation; both locations were associated with limestone outcrops that provide suitable roosting conditions for cave-dependent species. In contrast, the open-space foragers *Miniopterus magnater* and *Scotophilus kuhlii* were captured at low elevation, consistent with their preference for less cluttered flight paths. During the wet season, single individuals of the frugivorous bats *Macroglossus minimus* and *Cynopterus sphinx* were captured exclusively in the rice field area, likely reflecting opportunistic foraging movements rather than established local populations.

These spatially distinct occurrences indicate that Gunung Keriang functions as a heterogeneous landscape supporting both resident and transient bat species. The limestone karst provides critical roosting habitats, while adjacent rice fields offer temporally abundant food resources. The low capture frequencies of rare species likely reflect occasional foraging or dispersal movements, underscoring the importance of habitat mosaics in sustaining regional bat diversity and ecosystem services.

**Figure 4:**
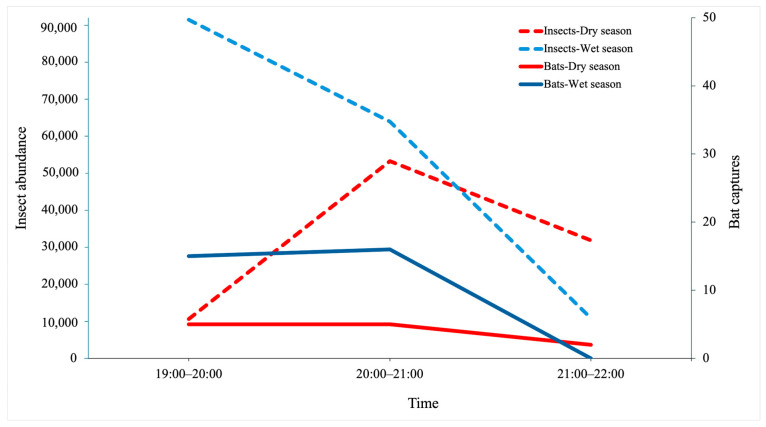
Temporal activity patterns of insects and bats during the dry and wet seasons.

The time of bat emergence in the dry season was from 19:00 until 21:00 while for wet season was from 20:00 until 21:00 (Figure 4). In the dry season, the bats emerged simultaneously with peak insect activity, whereas in the wet season, bats emerged later than insects.

**Figure 5:**
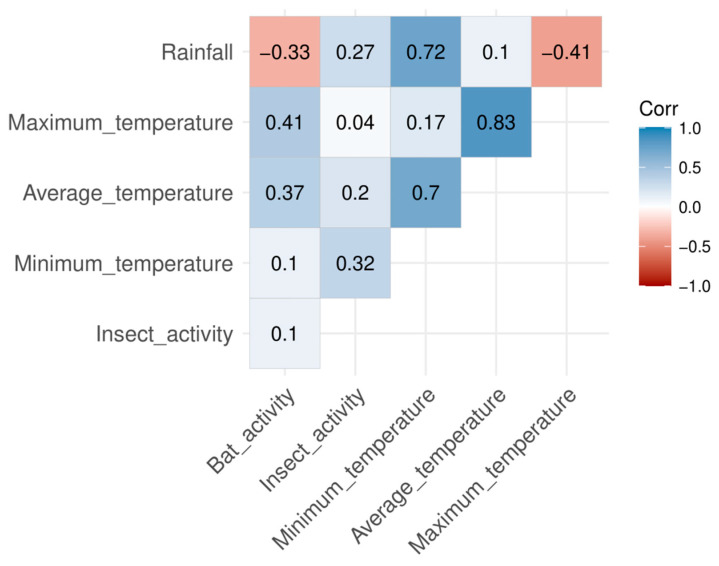
Correlogram of overall bat activity and insect activity associated with average temperature (°C) and rainfall (mm).

Based on the correlogram, the results show that the bat activity was positively correlated with the average temperature and negatively correlated with maximum temperature and rainfall (see diagnostic plot in Supplementary Material Table S1). The insect activity was positively correlated with minimum temperature and rainfall (Figure 5). The multiple linear regression model met the assumptions of linearity, homoscedasticity, independence and normality of residuals, as assessed using diagnostic plots (see diagnostic plot in Supplementary Material Figure S1) [29]. It is important to note that these patterns reflect associations rather than causation. While climatic factors appear linked with bat activity, our analyses cannot confirm a direct causal effect. These correlations suggest potential influences of microclimatic conditions on bat foraging behavior, which should be interpreted with caution.

**Figure 6:**
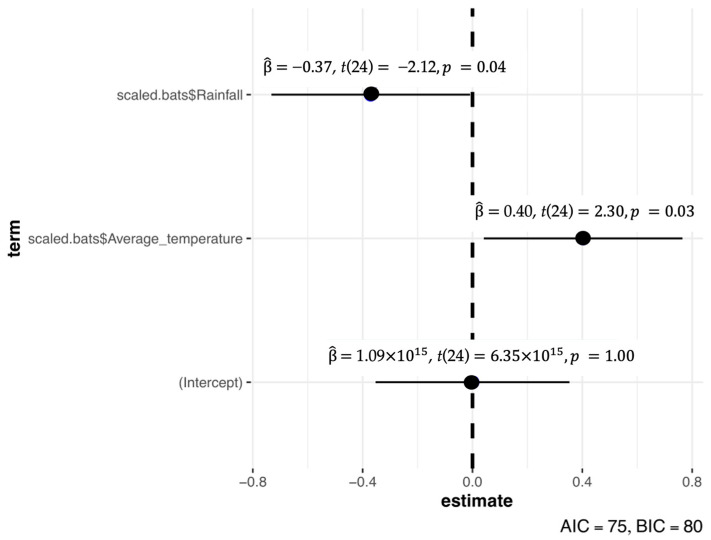
The coefficient values for the multiple linear regression model output. The *y*-axis label “scaled.bats” refers to the standardized bat activity variable used in the regression analysis.

The multiple linear regression model explained a moderate proportion of the variation in overall bat activity (R^2^ = 0.35; Figure 6). Minimum temperature had a significant positive effect on bat activity (standardized β = 0.40, *t*(24) = 2.30, *p* = 0.03), whereas rainfall showed a significant negative effect (standardized β = −0.37, *t*(24) = −2.12, *p* = 0.04). Average temperature was not retained as a significant predictor after model selection. Separate regression analysis for the five dominant bat species (*Rhinolophus pusillus*, *Myotis ridleyi*, *Hipposideros larvatus*, *Rhinolophus malayanus* and *Rhinolophus refulgens*) did not yield statistically significant models (all *p* > 0.05; see Supplementary Material Table S2) and were therefore discarded.

## 4. Discussion

This study relies on capture-based measures of bat activity, which are known to introduce sampling bias. Mist nets and harp traps tend to undersample fast-flying aerial insectivores and species that fly above the canopy or avoid obstacles. Consequently, our activity metrics may underestimate the presence and activity of these taxa. While the use of multiple trap types and temporal replication improves sampling coverage, our results should be interpreted with the understanding that they reflect the activity of species most likely to be captured, rather than the full spectrum of species active in the area.

This study provides one of the first insights into the relationship between insect abundance and bat activity in Malaysian rice field ecosystems. We found that insect composition and abundance varied with paddy phenology and season, but bat activity was influenced mainly by temperature and rainfall rather than by insect abundance. These results suggest that while food availability plays a role, local climatic conditions may exert a stronger influence on bat foraging behavior in this landscape.

We acknowledge that light traps selectively attract nocturnal, phototactic insects, which may lead to an overrepresentation of certain taxa, such as Coleoptera, in our samples. Other insect orders that are less phototactic or more active in different microhabitats may be underrepresented. While light traps are effective for monitoring pest populations and overall insect activity, our results should be interpreted with caution, recognizing that they reflect primarily the subset of insects attracted to light rather than the full diversity of the rice field insect community. Future studies combining multiple sampling methods could provide a more comprehensive assessment of insect diversity.

Coleoptera and Homoptera were the most dominant insect orders in the dry and wet seasons, respectively. The high abundance of Coleoptera, a hard-bodied prey, could favor larger bat species with strong bite force, such as *Hipposideros* and *Rhinolophus* spp., which are capable of handling such prey efficiently [30]. Previous dietary studies at Gunung Keriang [31] similarly revealed that larger insectivorous bats consume a broad range of insects, indicating opportunistic feeding linked to prey availability. In contrast, the wet-season dominance of Homoptera—particularly the brown planthopper *Nilaparvata lugens*—reflects the known seasonal pest outbreaks associated with high rainfall and humidity [32].

Although Homoptera fragments were rarely detected in prior diet analyses, this may reflect sampling limitations rather than true avoidance, underscoring the need for molecular diet studies with larger sample sizes. We acknowledge that our study did not conduct direct dietary analysis of bats; therefore, the predator–prey relationships discussed are inferred based on temporal overlap between bat activity and insect abundance, supported by existing literature on known bat diets. To confirm prey selection, particularly for taxa such as Homoptera, future research should incorporate molecular approaches such as eDNA or fecal metabarcoding, which would provide more direct and robust evidence of bat–insect interactions in this ecosystem.

Insect emergence peaked around 19:00–21:00, coinciding with peak bat activity. This synchrony suggests that bats respond to nightly prey pulses [33,34]. However, our correlation and regression analyses indicate that insect abundance did not directly predict bat activity. Instead, minimum temperature and rainfall were significant predictors, explaining 35% of the variation in bat activity. Bats likely adjust their foraging behavior to local weather conditions by avoiding heavy rain, which interferes with echolocation [35], and increasing activity during warmer, calmer nights [36].

The absence of a significant bat–insect abundance relationship may be due to several factors. First, both bats and insects were sampled independently, and temporal resolution may have been too coarse to detect real-time predator–prey responses. Second, artificial light, humidity, and moonlight, which are known to affect both insect attraction and bat activity [37,38], were not included as covariates. Finally, pooling species-level data into a single community index could obscure species-specific responses, as different bat guilds exploit different foraging niches [27]. Future studies should integrate acoustic monitoring with molecular diet data to better resolve these ecological links.

Based on our results, future molecular dietary analyses can be more strategically targeted. The consistent dominance of *Rhinolophus pusillus*, *Myotis ridleyi* and *Hipposideros larvatus*, together with their temporal overlap with peak insect emergence, suggests that these species are key candidates for molecular diet studies in rice agroecosystems. Similarly, the repeated occurrence of major rice pests such as *Chilo polychrysus* during the dry season and *Nilaparvata lugens* during the wet season highlights these taxa as priority targets for assessing bat-mediated pest suppression. Molecular approaches such as fecal metabarcoding or eDNA analysis should therefore focus on these bat-pest pairs to test specific predator-prey hypotheses generated by our observational data, rather than relying on broad community-level screening.

The detection of several rare and threatened species (based on the IUCN Red List of Threatened Species, *Rhinolophus convexus* is listed as data deficient [DD], *Hipposideros pomona* as endangered [EN], *Hipposideros halophyllus* as vulnerable [VU], *Myotis ridleyi* and *Hipposideros lekaguli* as near threatened [NT], and the remaining species as least concern [LC]) highlights the ecological importance of Gunung Keriang as a roosting and foraging habitat. The occurrence of rare bat species at specific elevations and habitat interfaces indicates that Gunung Keriang functions as a heterogeneous landscape supporting both resident and transient species. Limestone outcrops provide roosting opportunities for cave-dependent bats, while adjacent rice fields offer temporally abundant insect prey. The low capture numbers of these rare species likely reflect occasional foraging or dispersal movements rather than established local populations, emphasizing the importance of habitat mosaics in maintaining regional bat diversity. Consequently, conserving Gunung Keriang and its surrounding rice field matrix is crucial not only for bat conservation but also for sustaining ecosystem services such as natural pest regulation.

Our findings emphasize the ecological services provided by insectivorous bats in agroecosystems. Although we did not establish a direct quantitative link between bat activity and insect pest suppression, the observed temporal overlap between bat foraging periods and peaks in insect (including pest) availability suggests a potential regulatory role. These descriptive patterns provide valuable baseline information for understanding bat–insect interactions in rice agroecosystems, particularly in regions where such data remain limited. Promoting bat-friendly agricultural practices such as maintaining roosting habitats, reducing pesticide use and minimizing light pollution could enhance sustainable pest control in Malaysian rice fields.

Overall, this study contributes novel baseline data on bat–insect dynamics in tropical rice agroecosystems. While weather emerged as a stronger driver of bat activity than insect abundance, the consistent overlap in temporal activity between bats and pest insects underscores bats’ potential role in biological control. Integrating bat conservation into agricultural management can provide long-term ecological and economic benefits, supporting biodiversity and crop protection simultaneously.

## 5. Conclusions

This study provides valuable insights into the relationship between bat activity, insect abundance, and environmental factors in Malaysian rice field ecosystems. We recorded 27 bat species and 11 insect orders at Gunung Keriang, Kedah, highlighting the high biodiversity supported by this agroecosystem. *Rhinolophus pusillus* was identified as the most dominant bat species, while *Chilo polychrysus* and *Nilaparvata lugens* were the main insect pests during the dry and wet seasons, respectively.

Although bat activity overlapped with periods of high insect abundance, statistical analysis indicated that temperature and rainfall were the main factors influencing bat activity, suggesting that environmental conditions, rather than prey availability alone, may play a stronger role in shaping bat foraging behavior.

While our results are descriptive and do not provide direct quantitative evidence of pest suppression, the observed temporal overlap of bat and insect activity supports the possibility that insectivorous bats contribute to natural pest regulation in rice field ecosystems. Protecting bat habitats and reducing pesticide use could help maintain this potential natural pest control service. Integrating bat conservation into sustainable agricultural management may offer a cost-effective and environmentally friendly approach to pest control, benefiting both biodiversity and crop productivity. Future studies combining acoustic monitoring and molecular diet analysis would provide more direct evidence and deeper insights into bat–insect interactions in tropical agroecosystems.

## Figures and Tables

**Table 1:** Bats and insect pests captured at different growing phases of paddy for the dry and wet seasons.

Season/Phase	Bats			Insects			
	Number of Species Captured	Dominant Species (n/N)	Least Captured Species (n = 1)	Number of Orders Captured	Dominant Order (n/N)	Dominant Pest Species	Least Captured Pest Species
Dry/ Germination	15	*M. ridleyi* (158/609) *R. pusillus* (152/609)	*R. coelophyllus* *R. stheno*	10	Coleoptera (56,592/63,442) Hemiptera (3677/63,442)	*Chilo polychrysus* (Stemborer) *Nilaparvata lugens* (Brown planthopper)	*Oxya chinensis* (Grasshopper) *Gryllotalpa* *orientalis* (Mole cricket)
Dry/ Vegetative	17	*R. pusillus* (88/218) *H. cineraceus* (31/218)	*M. magnater* *H. bicolor* *M. muricola* *S. kuhlii*	10	Coleoptera (17,813/26,327) Dermaptera (2775/26,327)	*Chilo polychrysus* (Stem borer) *Nilaparvata lugens* (Brown planthopper)	*Gryllotalpa* *orientalis* (Mole cricket)
Dry/ Ripening	16	*R. pusillus* (271/517) *H. larvatus* (72/517)	*H. diadema* *H. kunzi* *H. lekaguli* *H. pomona* *R. marshalli*	11	Coleoptera (11,349/22,634) Homoptera (7235/22,634)	*Chilo polychrysus* (Stem borer) *Recilia dorsalis* (Zigzag leafhopper)	*Oxya chinensis* (Grasshopper)
Dry/ Harvesting	14	*R. pusillus* (68/268) *M. ridleyi* (62/268)	*H. pomona* *H. halophyllus*	7	Coleoptera (13,214/25,643) Hemiptera (11,448/25,643)	*Chilo polychrysus* (Stem borer) *Nilaparvata lugens* (Brown planthopper)	*Leptocorisa* *oratorius* (Rice ear bug) *Gryllotalpa* *orientalis* (Mole cricket)
Wet/ Germination	13	*R. pusillus* (100/197) *M. ridleyi* (24/197)	*H. armiger* *H. diadema*	8	Coleoptera (442/710) Homoptera (192/710)	*Recilia dorsalis* (Zigzag leafhopper) *Nilaparvata lugens* (Brown planthopper)	*Leptocorisa* *oratorius* (Rice ear bug) *Chilo polychrysus* (Stem borer)
Wet/ Vegetative	11	*R. pusillus* (75/167) *R. malayanus* (29/167)	*H. kunzi* *H. pomona*	8	Homoptera (64,626/65,368) Coleoptera (584/65,368)	*Chilo polychrysus* (Stem borer) *Nilaparvata lugens* (Brown planthopper)	*Oxya chinensis* (Grasshopper) *Gryllotalpa* *orientalis* (Mole cricket)
Wet/ Reproductive	14	*R. pusillus* (40/114) *R. malayanus* (14/114)	*H. diadema* *M. minimus*	10	Homoptera (3809/5450) Coleoptera (637/5450)	*Chilo polychrysus* (Stem borer) *Nilaparvata lugens* (Brown planthopper)	*Leptocorisa* *oratorius* (Rice ear bug) *Oxya chinensis* (Grasshopper)
Wet/ Ripening	19	*R. refulgens* (91/262) *H. larvatus* (43/262)	*H. bicolor* *H. kunzi* *H. halophyllus* *T. melanopogon* *C. sphinx*	7	Homoptera (3100/5676) Hemiptera (1541/5676)	*Chilo polychrysus* (Stem borer) *Nilaparvata lugens* (Brown planthopper)	*Oxya chinensis* (Grasshopper)
Wet/ Harvesting	12	*R. pusillus* (45/151) *R. malayanus* (32/151)	*H. lekaguli* *H. halophyllus*	8	Coleoptera (10,825/13,165) Homoptera (1274/13,165)	*Chilo polychrysus* (Stem borer) *Nilaparvata lugens* (Brown planthopper)	*Leptocorisa* *oratorius* (Rice ear bug)

n = number of individuals, N = number of total individuals.

## Data Availability

Data are contained within the article.

## Supplementary Materials

The following supporting information can be downloaded at https://www.mdpi.com/article/10.3390/biology15010069/s1. Figure S1: The conditions for multiple linear regression of overall bat activity and insect activity associated with minimum temperature (°C) and rainfall (mm); Table S1: Means, standard deviations, and correlations with confidence intervals of the correlation matrix of overall bat activity and insect activity associated with average temperature (°C) and rainfall (mm). Table S2: Values obtained in regression analysis. The *p*-value is calculated using the t-statistic from the T distribution (Pr(>|t|), the coefficient of determination (R^2^), the values of F and overall *p*-value came from the regression model.
