# Supplementary material for: Bat Community Response to Insect Abundance in Relation to Rice Phenology in Peninsular Malaysia
Source: Biology (Basel). 2025 Dec 30;15(1):69. doi: 10.3390/biology15010069 (PMC12785042; doi:10.3390/biology15010069)
Supplement: Supplementary file 1 [file biology-15-00069-s001.zip › Supplementary materials-Figure S1.pdf]

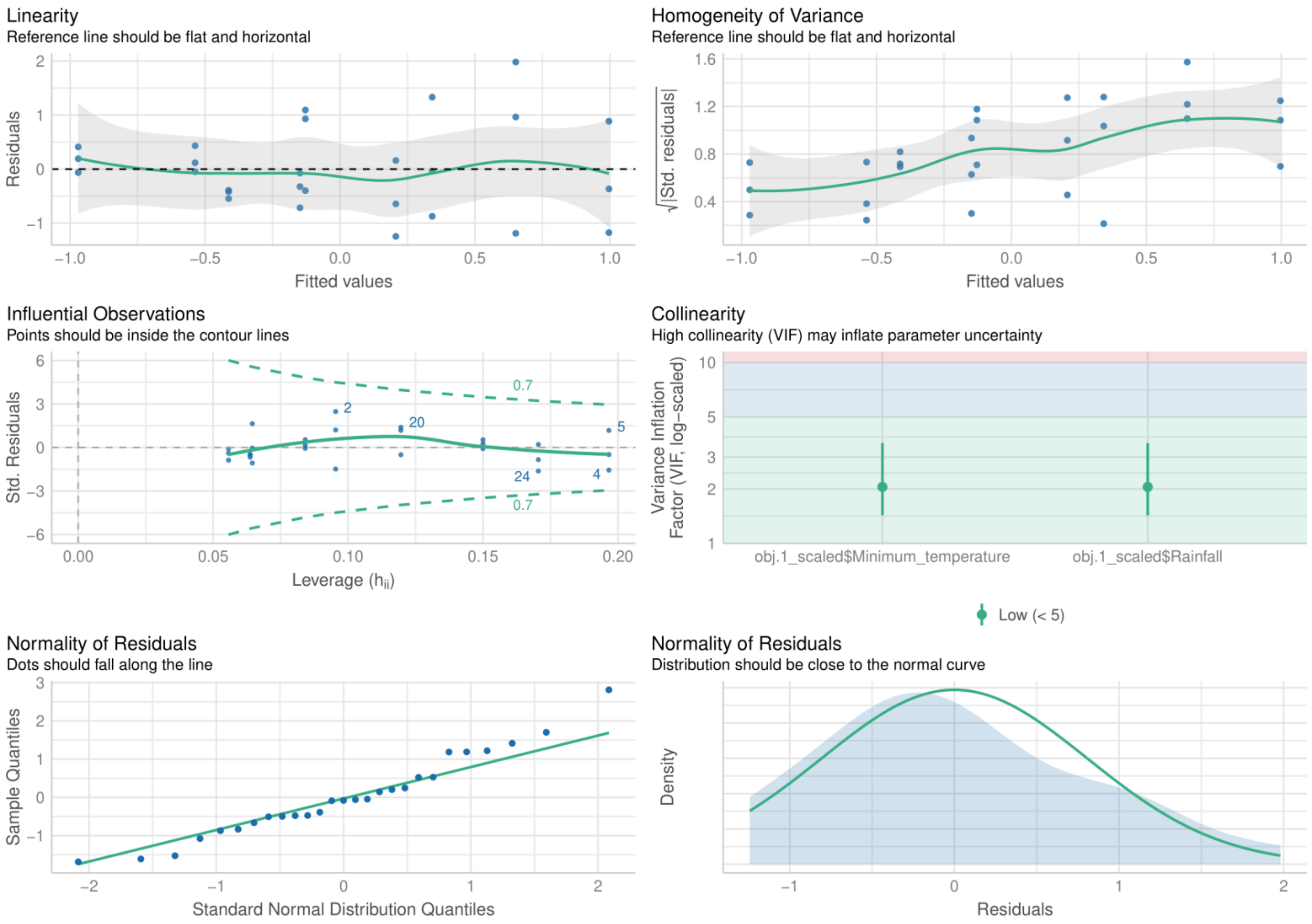

Figure S1: The conditions for multiple linear regression of overall bat activity and insect activity associated with minimum temperature(°C) and rainfall(mm).
