# Supplementary material for: Bat Community Response to Insect Abundance in Relation to Rice Phenology in Peninsular Malaysia
Source: Biology (Basel). 2025 Dec 30;15(1):69. doi: 10.3390/biology15010069 (PMC12785042; doi:10.3390/biology15010069)
Supplement: Supplementary file 1 [file biology-15-00069-s001.zip › Supplementary materials-Table S2.pdf]

Table S2: Values obtained in regression analysis. The *p*-value is calculated using the t-statistic from the T distribution (Pr(>|t|), the coefficient of determination (R^2), the values of F and overall *p*-value came from the regression model.

	**Variables**	**Pr(>|t|)**	**R^2**	**F**	***p*-value**
Rhinolophus pusillus					
	Insect activity	0.72	0.13	0.65	0.66
	Minimum temperature	0.70			
	Average temperature	0.69			
	Maximum temperature	0.67			
	Rainfall	0.54			
Myotis ridleyi					
	Insect activity	0.54	0.09	0.43	0.82
	Minimum temperature	0.78			
	Average temperature	0.79			
	Maximum temperature	0.83			
	Rainfall	0.51			
Hipposideros larvatus					
	Insect activity	0.15	0.16	0.79	0.57
	Minimum temperature	0.98			
	Average temperature	0.94			
	Maximum temperature	0.94			
	Rainfall	0.68			
Rhinolophus malayanus					
	Insect activity	0.50	0.07	0.33	0.89
	Minimum temperature	0.94			
	Average temperature	0.96			
	Maximum temperature	0.99			
	Rainfall	0.61			
Rhinolophus refulgens					
	Insect activity	0.70	0.13	0.60	0.70
	Minimum temperature	0.98			
	Average temperature	0.93			
	Maximum temperature	0.88			
	Rainfall	0.80			
