# Supplementary material for: Bat Community Response to Insect Abundance in Relation to Rice Phenology in Peninsular Malaysia
Source: Biology (Basel). 2025 Dec 30;15(1):69. doi: 10.3390/biology15010069 (PMC12785042; doi:10.3390/biology15010069)
Supplement: Supplementary file 1 [file biology-15-00069-s001.zip › Supplementary materials-Table S1.pdf]

Table S1: Means, standard deviations, and correlations with confidence intervals of the correlation matrix of overall bat activity and insect activity associated with average temperature(°C) and rainfall(mm).

Variable	M	SD	Insect activity	Minimum temperature	Average temperature	Maximum temperature	Rainfall
1. Bat activity	414.74	385.77					
2. Insect activity	8119.56	15339.92	.10				
			[-.30, .46]				
3. Minimum temperature	22.47	0.73	.10	.32			
			[-.29, .47]	[-.07, .62]			
4. Average temperature	28.56	0.62	.37	.20	.70**		
			[-.02, .66]	[-.19, .54]	[.43, .85]		
5. Maximum temperature	34.58	0.88	.41*	.04	.17	.83**	
			[.04, .68]	[-.35, .41]	[-.22, .52]	[.65, .92]	
6. Rainfall	219.69	128.37	-.33	.27	.72**	.10	-.41*
			[-.63, .06]	[-.13, .59]	[.46, .86]	[-.29, .46]	[-.69, -.04]

Note. *M* and *SD* are used to represent mean and standard deviation, respectively. Values in square brackets indicate the 95% confidence interval for each correlation. The confidence interval is a plausible range of population correlations that could have caused the sample correlation (Cumming, 2014). * indicates $p < 0.05$. ** indicates $p < 0.01$.
